# Undetected Common Mental Disorders in Long-Term Sickness Absence

**DOI:** 10.1155/2012/474989

**Published:** 2012-05-14

**Authors:** Hans Joergen Soegaard

**Affiliations:** Regional Psychiatric Services Central Denmark Region, Research Unit West and Centre for Psychiatric Research, 7400 Herning, Denmark

## Abstract

*Background*. Undetected Common Mental Disorders (CMDs) amongst people on sick leave complicate rehabilitation and return to work because appropriate treatments are not initiated. *Aims*. The aim of this study is to estimate (1) the frequencies of CMD, (2) the predictors of undetected CMD, and (3) the rate of return to work among sick listed individuals without a psychiatric disorder, who are registered on long-term sickness absence (LSA). *Methods*. A total of 2,414 incident individuals on LSA with a response rate of 46.4%, were identified for a two-phase study. The subsample of this study involved individuals registered on LSA who were sick-listed without a psychiatric sick leave diagnosis. In this respect, Phase 1 included 831 individuals, who were screened for mental disorders. In Phase 2, following the screening of Phase 1, 227 individuals were thoroughly examined by a psychiatrist applying Present State Examination. The analyses of the study were carried out based on the 227 individuals from Phase 2 and, subsequently, weighted to be representative of the 831 individuals in Phase 1. *Results*. The frequencies of undetected mental disorders among all sick-listed individuals were for any psychiatric diagnosis 21%, depression 14%, anxiety 4%, and somatoform disorder 6%. *Conclusions*. Undetected CMD may delay the initiation of appropriate treatment and complicate the rehabilitation and return to work.

## 1. Background

Common Mental Disorders (CMDs) impose suffering on and reduce quality of life of the individuals. They also place economic burdens on society, primarily due to indirect costs in regards to sickness absence, early retirement, and early death [1, 2]. In addition, depressive disorders significantly influence the outcome of comorbid medical illnesses such as cardiac diseases, diabetes, and cancer [3]. Furthermore, the emergence of a depression in an individual is likely to cause family dysfunction and risks of mental and physical illnesses among family members as well [4].

The burden of CMD may be even heavier than estimated in previous studies of this kind because CMDs are overlooked. This has been documented in primary care [5–12], in work places [13], in granting of disability pension [14], and among patient populations such as patients with, for example, chronic musculoskeletal pain [15], and in writing sick leave certificates [16–18]. Undetected mental disorders in primary care and sick leave certificates apply to the present study because the study is based on sickness absence and because sick leave certificates for the most part are certified in primary care.

The objective of the study of undetected CMD was to analyse the implications of undetected mental disorders in long-term sickness absence (LSA). The perspective was to provide an account of all new undetected mental disorders in LSA within one year by identifying all incident individuals on LSA within a well-defined region along with the application of methods to detect undetected psychiatric diagnoses.

## 2. Aim

On the basis of LSA, the aims were to (i) estimate the frequencies of undetected CMD: depression, anxiety, and somatoform disorders; (ii) identify sociodemographic predictors of CMD among individuals who were sick-listed minus a psychiatric sick leave diagnosis. (iii) identify sociodemographic predictors of return to work for CMD among individuals who were sick listed minus a psychiatric sick leave diagnosis.

## 3. Methods

### 3.1. Study Population

**Figure 1:**
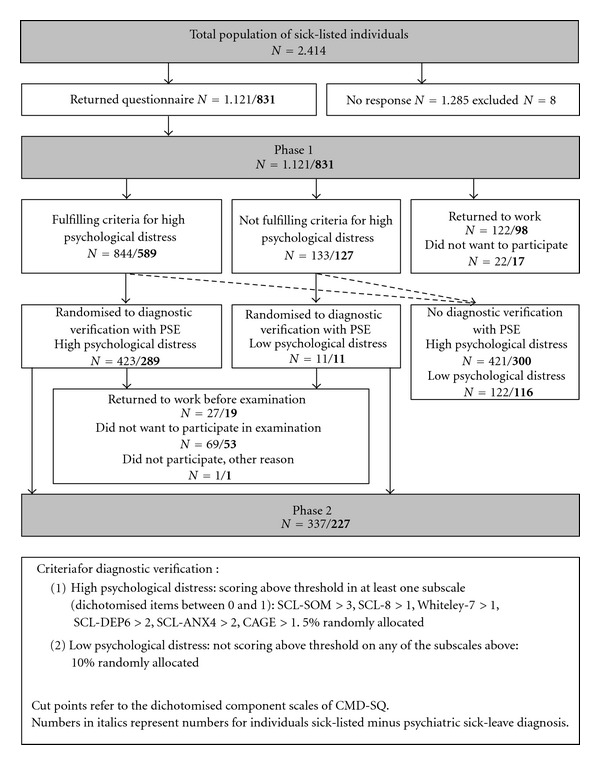
Flowchart of sick-listed individuals on LSA, target population, eligible individuals, selection criteria, and nonparticipation.

Figure 1 is a flowchart representing (1) the total of 2,414 individuals that were sick listed on LSA within one year, (2) the selection procedures to reach the eligible individuals for this study, and (3) the categories of nonparticipation. LSA was defined as continuous sickness absence exceeding eight weeks. The study took place in six Danish municipalities with a total of 118,000 inhabitants of whom 50% were living in the urban municipality of Herning. In Denmark, the social services are responsible for sickness benefits after two weeks of continuous sickness absence. Due to this setup of sick individuals receiving benefits from the social services, it was possible to identify *all* new coming individuals on LSA who had their first day of sickness absence between the 30th of August 2004 and the 29th of August 2005. Furthermore, this registration facilitated the identification of individuals entering LSA. On a weekly basis, the Danish social services provided this study with information regarding sick listed individuals based on public registers. Irrespective of their reasons for being sick-listed, the 2,414 individuals comprise individuals who were sick-listed from full-time work, part-time work, or adjusted work as well as unemployed individuals who became ill and changed registered status from receiving unemployment benefits to receiving sickness benefits. If an individual was registered as being on LSA more than once within the year current for this study, only the first period was registered. The following individuals were excluded: individuals below 18 years at the day when the sickness absence period exceeded eight weeks, individuals on maternity leave, and non-Danish speakers.

### 3.2. Two-Phase Study and Definition of Concepts

The study was carried out as a two-Phase investigation. In Phase 1, all 2,414 sick listed LSA individuals were asked to fill out a screening questionnaire. Out of the 2,414 individuals, 1,121 (46.4%) responded, and, subsequently, 831 individuals presented without a psychiatric sick leave diagnosis. The individuals of primary concern in this study were these 831 individuals minus a psychiatric sick leave diagnosis referred to as *sick listed minus a psychiatric sick leave diagnosis *(*MPSD*) (shown in bold in Figure 1). 290 individuals were sick listed *plus a psychiatric sick leave diagnosis *(*PPSD*). In Phase 2, MPSD individuals, who underwent a psychiatric examination and presented with a psychiatric diagnosis, constitute a group referred to as *verified psychiatric diagnosis *(*VPD*). For individuals sick listed MPSD and PPSD, the total number of VPD was 188.

### 3.3. Phase 1

The screening for mental disorders in Phase 1 was carried out by means of the subscales in Common Mental Disorders-Screening Questionnaire (CMD-SQ) according to the criteria mentioned in Figure 1 [19]. Along with the return of the questionnaire, written informed consent was given to participate in the study. In Phase 1, sick leave diagnoses were provided from social services.

### 3.4. Phase 2

Prior to Phase 2, 122/**98** individuals returned to work and, thus, were no longer on LSA and, thereby, no longer eligible to participate in the study. In addition, 22/**17** did not want to participate any further (Figure 1). This resulted in 844/**589** individuals who had scored at a *high level of psychological distress* on the initial questionnaire of Phase 1. Half of this group was, by a research assistant, randomly allocated to Phase 2 (423/**289**). Furthermore, in order to ensure that an adequate number of individuals with low scores in the subscales of CMD-SQ were taken out for a diagnostic verification, 10% of the individuals who had not returned to work and who presented with a *low level of psychological distress* were randomly allocated to a psychiatric verification. This group turned out to be as low as 11/**11** individuals. Following, it appears from Figure 1 that along the process some individuals returned to work before a psychiatric examination could be arranged, others did not want to participate in the examination, and 1 did not participate for another reason. Finally, Phase 2 constituted 337/**227** individuals. After being allocated to the psychiatric examination, the individuals gave informed written consent to participate in an examination as well as consent to inform their general practitioners and social services about the results of the examination.

### 3.5. Data

#### 3.5.1. Verified Psychiatric Diagnoses (VPD)

VPD was identified for MPSD and PPSD by means of a psychiatric examination by a psychiatrist (the investigator HJS) who made use of the computerised SCAN version 2.1, programme version 1.0.4.6, Present State Examination as the gold standard (ICD-10 diagnoses) [20]. SCAN covers somatisation, anxiety disorders, affective disorders, dependence, and psychotic disorders. Disorders not covered by SCAN were diagnosed by the investigator (HJS) according to ICD-10 [21]. The psychiatric examinations were carried out by the investigator without any knowledge of the screening results, and they were conducted as quickly as possible subsequent to the participants having exceeded eight weeks of sickness absence, 10% within 19 days, 50% within 27 days, 90% within 44 days, and all within 68 days.

#### 3.5.2. Return to Work

The rate of return to work was analysed by means of survival methods by which the observation period was defined as the period from the first day of entering LSA until the payment of sickness benefits was stopped. An event was defined as return to normal full-time work, normal part-time work, and for unemployment if the registered sickness benefits status changed to unemployment benefits status. Normal part-time work was included as an event since this was often planned as a gradual return to work under normal conditions. Other reasons for terminating sickness benefits were defined as censoring. The dates for the beginning and termination of sickness benefits were recorded from social services registers.

#### 3.5.3. Sociodemographic Data

In addition to CMD-SQ, the questionnaire contained questions about sociodemographic characteristics.  Table 1 shows the categories and frequencies of the sociodemographic variables for individuals who were sick listed MPSD.

#### 3.5.4. Common Mental Disorders Screening Questionnaire, CMDQ-SQ

CMD-SQ is a symptom scale with six subscales concerning mental symptoms related to the diagnostic categories of depression, anxiety, somatoform disorders, and alcohol dependence [19]. The items are primarily derived from SCL-90/SCL-92 [22, 23]. SCL-SOM (12 items) covers somatisation [23], SCL-8 (8 items) emotional distress in general [24], SCL-ANX4 (4 items) anxiety, [19], and SCL-DEP6 (6 items) depression [19]. Some items belong to more subscales. In addition, the 7-item subscale, Whiteley-7, covers illness worry and conviction, and it is derived from the Illness Behaviour Questionnaire [25, 26]. Finally, the 4-item subscale CAGE [27] covers alcohol dependence.

CMD-SQ consists of 37 items of which 36 were relevant for this study. Items 1–32 are scored 0 to 4 on 5-point Likert scales, whereas items 33–36 are dichotomised items. Higher score indicate higher severity of symptoms.

A 13-item dichotomised component scale, SCL-8AD, composed of the items in SCL-ANX4, SCL-8, and SCL-DEP6 was created by dichotomising the items between 0 and 1. This scale turned out to have the best predictive properties when it came to the identification of CMD, for which reason it was used in the weighted analyses [28]. Furthermore, the screening criteria, indicated in Figure 1, were also based on dichotomised component scales of the other subscales of CMD-SQ.

#### 3.5.5. Sick Leave Diagnoses

Data about sick leave diagnoses were obtained from social services records in the form of transcriptions from sick leave certificates, discharge records, other medical documents, and in the form of declarations from sick-listed individuals. The sick leave diagnoses were based on the diagnostic information which was available to social services up to three months after the first day of sickness absence. The sick leave diagnoses were coded as ICPC diagnoses by the investigator (HJS) [29]. The only differentiation of sick leave diagnoses was whether the individual had a psychiatric sick leave diagnosis or not.

### 3.6. Statistical Analyses

The analyses were carried out in Phase 2 and made representative of Phase 1 by weighting [30]. As Phase 2 consists of subgroups with different variance, the confidence intervals were calculated by jackknife procedures [30]. The frequencies of verified CMD among all sick-listed individuals were analysed in regards to all 227 individuals in Phase 2 and weighted up to the 1,121 individuals in Phase 1. With regard to the frequencies of CMD among individuals minus a psychiatric sick leave diagnosis, the analyses were carried out on the 227 individuals in Phase 2 and weighted up to the 831 in Phase 1. Finally, the estimation of the frequencies of undetected CMD among individuals with a verified diagnosis were based on all the 188 individuals in Phase 2 who presented a verified diagnosis whether sick-listed PPSD or MPSD.

The socio-demographic predictors of VPD were estimated by weighted logistic regression based on the 227 individuals in Phase 2 sick-listed MPSD. This was also the case for the uncontrolled rates of return to work which was analysed by weighted Poisson regression and the socio-demographic predictors of return to work by weighted Cox-regression. The multivariable logistic regressions and Cox-regression were reduced by the forward stepwise procedure. This procedure was continued until a significant change in the log likelihood function reached the 5% level. To analyse the differential effects, interaction variables were created as VPD *socio-demographic characteristic. The confidence limits (CI) were estimated by the jackknife procedure in Phase 2 [31].

The statistical analyses were performed by STATA 10.0.

The study was approved by the local ethics committee, but was not found to be within the framework of the ethic committees (The Ethic Committee for Ringkjøbing, Ribe, and Sønderjylland counties ref. number 2607-04). Moreover, the study was approved by the Danish Data Protection Agency. The ethical considerations were discussed in a previous paper [17].

## 4. Results

### 4.1. Frequencies of Undetected Psychiatric Diagnoses

Table 2 shows that among all sick-listed individuals the frequencies of undetected mental disorders were as follows: any psychiatric disorder 21.4%, depression 14.2%, anxiety 4.4%, and somatoform disorder 6.4%. In addition the frequencies among individuals sick-listed MPSD and among individuals with a VPD are presented.

### 4.2. Predictors of Common Psychiatric Diagnoses

Table 3 illustrates that the Female gender was a significant predictor of depression and Female gender and Employment, others were significant predictors of somatoform disorder.

### 4.3. Return to Work

The rate of return to work was highest by a rate of 118.5 individuals/1000 sick-listed individuals/30 days for individuals who did not have a VPD. This was followed by anxiety (101.7), depression (60.8), and somatoform disorder (41.2) (not shown in tables).

Table 4 shows the result of a multivariable weighted Cox regression indicating the hazard rate ratios of return to normal work. The reference group for the psychiatric disorders depression, anxiety, and somatoform was no psychiatric diagnosis. Depression showed a significantly lower rate of return to work and anxiety showed a significantly higher rate. Somatoform disorder showed a significantly higher rate of return to work except for individuals who were white collar/civil servant where the rate was significantly lower.

## 5. Discussion

### 5.1. Perspective of the Study

The perspective was to detect all CMD by taking the following issues into account: (1) the *identification of all sick-listed individuals* and (2) the *detection of undetected mental disorders *among sick-listed individuals. The unique *identification of all sick-listed individuals* is, in contrast to non-Scandinavian countries, possible in Denmark because compensation for sickness absence beyond two weeks is paid for by the social services and, therefore, based on entries in public registers. Prior to the present study, a Norwegian study [32] within the field of LSA was carried out. This study was also based on national registers for sickness benefits; however, it did not apply methods for the *detection of undetected mental disorders* as the diagnoses were based on the sick leave diagnoses. In other studies, methods for the detection of mental disorders have only been applied in selected samples [5, 13–16, 18]. A single study has taken both issues into consideration making a comprehensive account for the frequencies of mental disorders in the field of LSA possible [17]. The present study, which focuses specifically on individuals with undetected CMD, is based on the cohort of the latter study.

### 5.2. Frequencies of Psychiatric Diagnoses

Among individuals with a VPD, the frequencies of undetected psychiatric diagnoses were 44%, 40%, 30%, and 87%, respectively. These frequencies are in accordance with studies in primary care showing that less than 50% of the individuals with a mental disorder were detected [5–12]. These studies agree that it is the fairly well-functioning individuals who are undetected as these individuals experience less severe symptoms; they have a higher social status, and a higher level of education. Furthermore, the individuals with undetected CMD only mention somatic symptoms as the reason for GP contact [6–12]. The same explanation may be applicable to LSA since the major part of sick leave diagnoses relies on the assessments of general practitioners. However, more factors may be involved. One factor may be that the primary care physicians to a high degree rely on functional rather than diagnostic criteria. Another factor may be that the physicians only diagnose a patient with a psychiatric disorder if they consider the patient to be in need of psychiatric medical treatment [7]. In addition, the patients' resistance against diagnosis may be of importance which also applies to the patients' willingness to treatment and adherence to treatment [7, 33]. It is also probable that diagnostics in primary care to a higher degree is based on a psychosocial approach than the more biomedical approach in ICD-10 or DSM-IV. This hypothesis is supported by a Swedish study that has identified psychosocial stressors as indicators in early detection of depression [32]. The differences of this study compared to others may also derive from a methodological bias, as the sick leave diagnoses originated from the beginning of the sickness absence period, whereas the verified diagnoses were established after eight weeks of sickness absence, and it is probable that some disorders have evolved after the sick listing.

### 5.3. Predictors of Verified Psychiatric Diagnoses

Female gender was the only predictor of depression. The two categories female gender and other employment were predictors of somatoform disorder. This is in accordance with population studies [34–41]. In addition, population studies have found the risk of mental disorders decreasing with increasing age [34, 35, 37, 38, 40]. Furthermore, population studies have found that urbanity, living alone, and unemployment are associated with high frequencies of mental disorder [34–44]. As it appears, the number of predictors was lower in the present study when compared to population studies. The reason for this may be that the present study focused on incident individuals on LSA, whereas population studies dealt with prevalence. Another explanation may be that this study was controlled for each of the socio-demographic characteristics. Finally, the lower number of predictors may be due to the fact the individuals with mental disorders in the cohort of MPSD have newly developed mental disorders. Consequently, the study may indicate that female gender may be a predictor of the development of depression, whereas socio-demographic indicators such as living alone and unemployment may be a consequence of having developed a mental disorder. It is important to emphasise that the study design does not guarantee that individuals without a psychiatric sick leave diagnosis at the beginning of the study have not previously had a mental disorder. However, it is likely that individuals with newly developed mental disorders occur more frequently in this subsample.

### 5.4. Return to Work

Individuals without *verified psychiatric diagnosis* were shown in the uncontrolled analyses to have the highest rates of return to work, whereas individuals with verified depression and verified somatoform disorder had the lowest. This finding is in agreement with other studies which show that the duration of sickness absence for individuals with mental disorders is comparable with that of chronic disabling conditions [45–48]. As it was the case for predictors of mental disorders, the predictors of return to work are relatively few in numbers in this study compared to other studies. Other studies indicate that increasing age is a predictor of long-term sickness absence [18, 49–55]. This is contrary to the findings in this study. The contrast between this study and others may reflect the possibility of retiring at the age of 60 years in Denmark, and it is likely that the healthiest individuals are the ones to persist in the labour market. In other studies, unemployment, as in this study, is associated with long-term lower return to work [50, 52, 53]. Most studies indicate that females have a higher number of sickness absence periods than men, whereas males have longer period than females [16, 18, 49, 51, 53–56]. In this study, however, gender was of no significance. Employment as a skilled worker was a strong predictor of a high rate of return to work. An explanation could be that blue-collar workers can often work despite a minor mental problem, whereas a similar problem may cause sickness absence for white-collar workers. However, in this study this was only the case when white collar workers had a somatoform disorder. Otherwise, they showed a significantly higher rate of return to work. The discrepancies with other studies may be explained by the fact that the individuals in this study had newly developed mental disorders. This issue needs further clarification, but if it is so, rehabilitation and return to work may be somewhat different with newly established disorders compared to more developed disorders.

### 5.5. Strengths and Limitations

One of the strengths of this study is that it accounts for all sick-listed individuals in a population without incomplete coverage. Another strength is that a subgroup of individuals had their psychiatric diagnoses verified.

The *total nonresponse* (53.6%) in Phase 1 may bias the generalisability with regard to all the 2,414 individuals. Firstly, men were represented at a lesser frequency among the participants than among the nonparticipants. Secondly, the participants were older than among the nonparticipants.

The delay concerning the completion of the psychiatric examinations may have induced a bias since the mental disorder could have developed after the sick-listing. This will overestimate the incidence of undetected CMD at the beginning of the sickness absence period. However, the presence of CMD, whether it presented itself before or after the beginning of the sickness period, may still influence the rehabilitation process which usually begins six to eight weeks after the sick-listing.

With regard to the estimation of the frequency of undetected disorders, the methodology in the present study is different from studies within primary care. In the studies of primary care the GPs diagnosed the patients under standardised conditions, whereas the information with regard to sick leave diagnoses in this study was gathered in a less standardised way, from medical documents. The less standardised conditions may lower the reliability of the sick leave diagnoses. However, the validity may be higher than in the studies of primary care due to the fact the standardised conditions increase the GPs awareness of giving a psychiatric diagnosis. Consequently, the present study does to a higher degree reflect the ordinary clinical setting.

The ICPC coding of sick leave diagnoses was done by the investigator which may have biased the coding as the research questions was known by the investigator [29]. This is unlikely, however, due to the fact that the information was gathered from medical records. Some information specifying a sick leave diagnosis may have been lost, and, therefore, some sick leave diagnoses may have been coded as symptom diagnoses even if a specific somatic disorder was present. Moreover, the study did not include a diagnostic verification of somatic diagnoses. Finally, some individuals who at the time of the coding of sick leave diagnoses were still under diagnostic examination for a somatic disorder may later have gotten a verified somatic diagnosis. Consequently, some individuals with a specific somatic diagnosis may have been registered with unspecific somatic symptom diagnoses. With respect to this study the only differentiation in the sick leave diagnoses was between somatic and psychiatric diagnoses. It is unlikely that somatic disorders were coded as psychiatric disorders, whereas it is more likely that psychiatric disorders were coded as unspecific somatic disorders. Consequently, a possible misclassification is not of much concern for this study.

The models for interaction between VPD and the predictors implied a large number of significance tests for which reason some of the significant associations may have occurred by chance. However, the 95% confidence limits were for all results far from 1 for which reason this bias in unlikely.

## 6. Conclusions

Among all sick-listed individuals on LSA, 21% had an undetected mental disorder. Females had significantly increased risks of CMD compared to males. Individuals who were employed as skilled workers had a significantly higher rate of return to work. Unemployment had a significantly decreased rate of return to work. The predictors of a CMD and return to work are somewhat different in this study compared to other studies. This may indicate that rehabilitation back to work for newly established mental disorders should be planned differently compared to more developed mental disorders. Undetected CMD is considered a serious health problem as it may delay the initiation of an appropriate treatment and complicate the rehabilitation and return to work. The rehabilitation of an individual with a somatic disorder may be more complex if it is comorbid with a mental disorder. For this reason, it is recommended that all sick leave certificates entail information regarding all comorbid disorders, and it is recommended that sick-listed individuals should be diagnosed according to ICPC after eight weeks of continuous sickness absence [29].

## Figures and Tables

**Table 1:** Frequencies of sociodemographic characteristics including 95% confidence intervals (CI) for each frequency among individuals minus a psychiatric sick leave diagnosis.

Sociodemographic characteristics	Frequency %	95% CI
*Gender*		
Men	47.9	44.5–51.3
Women	52.1	48.7–55.5
*Age*		
−29 years	10.8	8.6–12.9
30–39 years	19.2	16.5–21.9
40–49 years	29.5	26.4–32.6
50–59 years	33.6	30.4–36.8
60 years +	6.9	5.1–8.6
*Municipality*		
Urban	50.7	47.3–54.1
Rural	49.3	45.9–52.7
*Household*		
Living with partner	81.0	78.3–83.7
Living without partner	19.0	16.3–21.7
Living with children below 18 years	48.5	45.1–52.0
Not living with children below 18 years	51.5	48.0–54.9
*General education*		
General: primary and lower secondary school	72.9	69.8–76.0
General: more than primary school	27.1	24.0–30.2
*Specific education*		
Unskilled worker	28.9	25.5–31.6
Skilled worker	50.7	47.2–54.1
Theoretical ≤ 4 years beyond primary school	17.1	14.6–19.7
Theoretical > 4 years beyond primary school	1.7	0.8–2.6
Other specific education	3.6	2.4–4.9
*Employment*		
Self-employed	8.4	6.5–10.3
White-collar/civil servant	34.5	31.2–37.7
Skilled worker	11.2	9.1–13.5
Unskilled worker	28.1	25.1–31.2
Other	17.7	15.1–20.3
*Employment situation*		
Full-time employment	71.2	68.1–74.3
Part-time employment	17.4	14.8–20.0
Unemployed	11.4	9.2–13.6

**Table 2:** Frequencies of undetected CMD in the entire sick-listed population, in individuals minus a psychiatric sick leave diagnosis, and in individuals with a verified psychiatric diagnosis including 95% confidence intervals (CI) for each proportion.

Psychiatric diagnosis	Weighted frequencies of CMD among all sick-listed individuals based on Phase 2	95% CI	Weighted frequencies of CMD among individuals minus psychiatric sick leavee diagnosis	95% CI	Weighted frequencies of CMD among individuals with a verified diagnosis	95% CI
	*N* = 337 %		*N* = 227 %		*N* = 188 %	
Any psychiatric diagnosis	21.4	17.5–25.9	30.2	24.8–36.2	44.4	37.4–51.6
Depression	14.2	11.0–18.2	19.9	15.4–25.3	40.2	32.3–48.7
Anxiety	4.4	2.7–7.2	6.4	3.9–10.3	30.0	19.3–43.5
Somatoform disorder	6.4	2.9–13.6	8.9	4.1–18.4	86.5	73.5–93.7

**Table 3:** Predictors of verified depression, anxiety, and somatoform disorder among individuals sick-listed minus psychiatric sick leave diagnosis including 95% confidence intervals (CI) for each odds ratio (OR). ORs are significant when the 95% confidence limits not include 1.

Psychiatric diagnosis	Risk factor	OR	95% CI
Depression			
	Female	2.53	2.42–2.65
	Constant	0.15	0.14-0.15

Anxiety		—	—

Somatoform disorder			
	Female	3.20	2.63–3.89
	Employment—Other	5.27	4.81–5.77
	Constant	0.04	0.03–0.05

**Table 4:** Cox-regression indicating predictors of return to work for individuals minus psychiatric sick leave diagnosis including 95% confidence intervals (CI) for each hazard rate ratio (HR). HRs are significant when the 95% confidence limits not include 1.

Category	HR	95% CI
Depression	0.59	0.54–0.64
Anxiety	1.50	1.29–1.74
Somatoform disorder	1.43	1.18–1.74
White collar/civil servant	1.40	1.36–1.44
Somatoform * White collar/civil servant	0.07	0.03–0.19
Employment-Skilled worker	2.25	2.11–2.39
Age 60 years +	1.94	1.77–2.13
Unemployed	0.33	0.28–0.38
